# Expression levels of T cell-related immune factors and their correlation with thyroid function in Graves’ disease with varied serum iodine status: insights into immunopathogenesis

**DOI:** 10.3389/fendo.2025.1623881

**Published:** 2025-08-15

**Authors:** Lilan Wang, Zixuan Ru, Shengnan Gao, Na Lv, Kerou Li, Hong Qiao

**Affiliations:** Department of Endocrinology, Second Affiliated Hospital of Harbin Medical University, Harbin, China

**Keywords:** iodine excess, T cell subsets, cytokines, thyroid function, Graves’ disease hyperthyroidism

## Abstract

**Objective:**

Measurement of Serum Iodine Concentration (SIC) in Newly Diagnosed Adult Graves’ Disease (GD) Patients with Hyperthyroidism and Healthy Controls: Investigating Cytokine Expression Profiles and Their Correlations with Thyroid Function Across Diverse Iodine Nutritional Status.

**Method:**

Patients newly diagnosed with GD hyperthyroidism from September 2024 to February 2025 at our institution were enrolled. Serum samples were collected for SIC measurement using arsenic-cerium catalytic spectrophotometry. Serum cytokine levels of 12 Th cell-related cytokines were quantified via LEGENDplex™ Human Th Cytokine Panel, and thyroid function was assessed by electrochemical immunoassay. Participants were stratified into three groups based on WHO iodine status criteria: iodine deficiency (<45 μg/L), adequate iodine (45-90 μg/L), and iodine excess (>90 μg/L). Pearson/Spearman correlation analyses were performed to evaluate associations between cytokine profiles and thyroid function parameters across subgroups.

**Results:**

Based on the inclusion and exclusion criteria, a total of 75 subjects were enrolled in this study. The SIC was 148.62 ± 17.63 μg/L (GD I group), 72.33 ± 12.08 μg/L (GD II group), and 75.24 ± 7.94 μg/L (NC group), respectively, with statistically significant differences among the three groups (P<0.001). In GD patients, SIC showed a positive correlation with TRAb levels (r = 0.136, P<0.001). Serum concentrations of IL-6, IL-4, IL-5, IL-13, IL-2, IFN-γ, TNF-α, IL-17A, and IL-22 in GD patients were significantly higher than those in the NC group, with the GD I group demonstrating notably elevated IL-6 levels compared to the GD II group (P<0.05). Correlation analysis revealed positive associations between IL-6 and SIC, TRAb, and IL-17A (r = 0.114, 0.105, 0.214; P < 0.05), while no correlations were observed with FT3, FT4, TSH, TPOAb, or TgAb levels (P > 0.05). Similarly, IL-17A exhibited positive correlations with SIC and IL-6 (r = 0.130, 0.214; P < 0.05), but showed no significant associations with FT3, FT4, TSH, TPOAb, or TgAb concentrations (P > 0.05).

**Conclusions:**

1. Serum cytokine levels (including IL-6, IL-9, IL-17A, IL-17F, and IL-22) exhibited significant differences between healthy subjects and patients with newly diagnosed hyperthyroid Graves’ disease (GD) under varying serum iodine concentration (SIC). 2. In newly diagnosed hyperthyroid GD patients, serum IL-6 demonstrated positive correlations with SIC, TRAb, and IL-17A (all P < 0.05), while IL-17A showed positive correlations with SIC and IL-6 (P < 0.05). 3. In GD patients with elevated SIC, cytokines IL-17A and IL-6 may contribute to pathogenic processes in hyperthyroid GD.

## 1 Introduction

**Figure 1:**
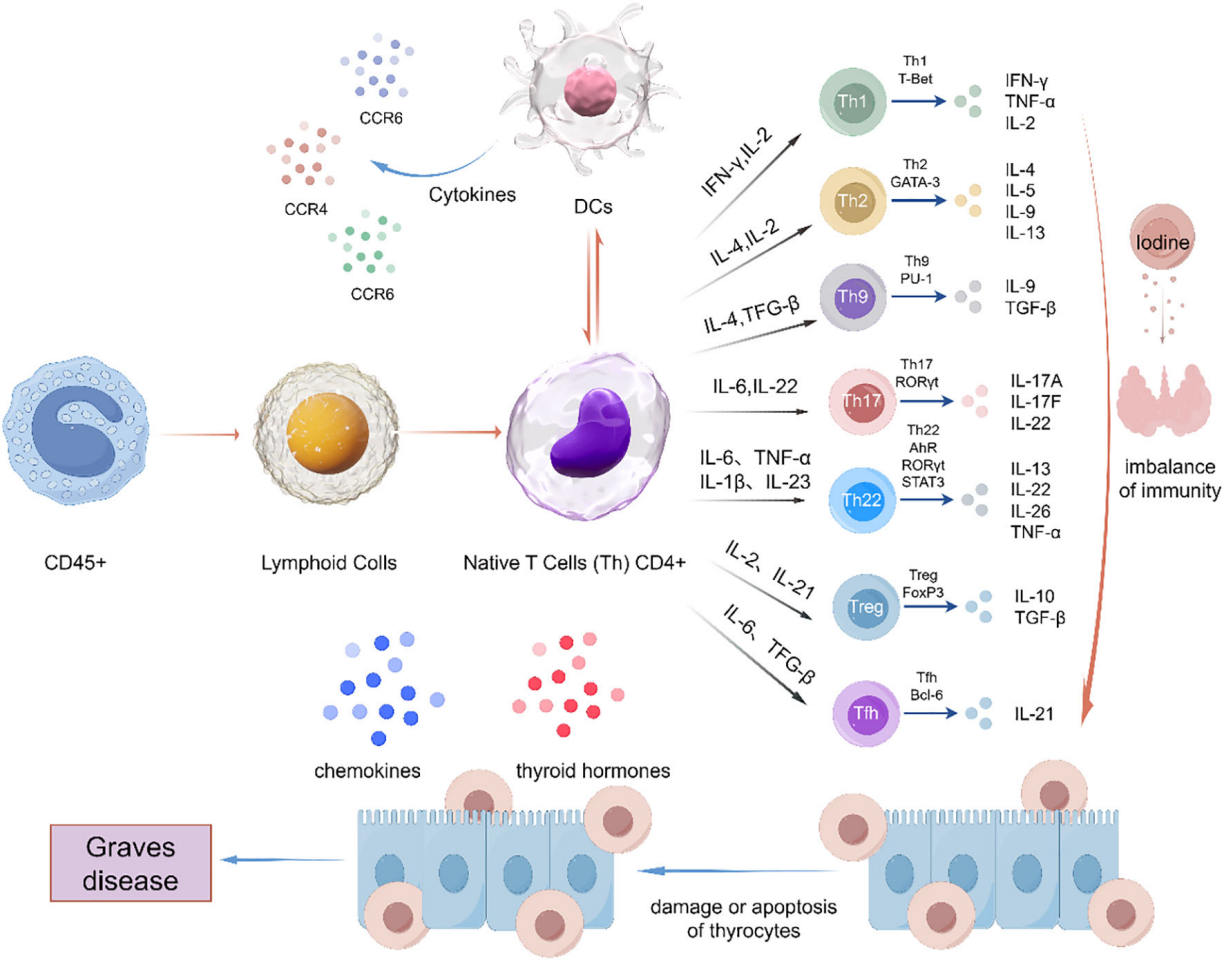
Immunopathogenesis of Graves’ disease. Within the thyroid microenvironment, dendritic cells present thyroid autoantigens to naïve T cells. Under the impact of the localized cytokine milieu and costimulatory signals, these T cells differentiate into diverse effector subsets (Th1, Th2, Th17, Th9) as well as regulatory T cells (Tregs). Pathological dysregulation of Th subset homeostasis, specifically the imbalance between Th17 and Tregs and the abnormal activation of follicular helper T cells (Tfh), triggers autoimmune cascades through three fundamental mechanisms: Firstly, the production of subset - specific cytokines (IFN-γ/IL-17/IL-21) which heighten thyrocyte HLA-II expression; secondly, the production of TRAb with the assistance of B cells; thirdly, direct cytotoxicity through the secretion of perforin and granzyme. This three-pronged immunological disruption ultimately leads to the hyperfunction of thyroid follicular cells and the progressive autoimmune destruction that is characteristic of GD.

Graves’ disease (GD) is an organ-specific autoimmune disorder of the thyroid driven by T cells and represents the leading cause of hyperthyroidism in adults, accounting for around 80% of cases. Despite its rising prevalence worldwide, the immunopathological mechanisms underlying GD remain not entirely understood understood (1). The disease results from interactions between genetic predisposition and environmental triggers (e.g., viral infections, dysregulated iodine intake), disrupting immune tolerance. This encourages the production of thyroid autoantibodies and abnormal T cell responses. Pathogenic CD4^+^ T cells proliferate and infiltrate thyroid tissues, releasing pro-inflammatory cytokines that damage thyroid epithelial cells and destabilize the immune microenvironment, driving GD pathogenesis (**Figure 1**).

Environmental factors play a significant role in triggering and worsening thyroid autoimmunity, with abnormal iodine consumption being a key external factor. The existing understanding of how excessive iodine worsens autoimmune thyroid disorders involves several mechanisms (2): (1) Ultrastructural alterations in thyroid cells: Iodine primarily induces thyroid epithelial cell damage, triggering the release of autoantigens (e.g., thyroglobulin, Tg) and proinflammatory cytokines (IL-1α, IL-6, IL-8), which perpetuate thyrocyte injury through feedforward inflammatory loops; (2) Enhanced immunogenicity of iodinated Tg: Iodine conjugation generates iodinated Tg (I-Tg), exhibiting superior immunostimulatory capacity compared to native Tg; (3) MHC class II antigen aberrant expression: Excessive iodine stimulates thymic remodeling and modulates immune cell development/function (T/B cells, macrophages, dendritic cells); (4) Augmented antigen presentation: Iodine potentiates macrophage phagocytosis of Tg and promotes thyroid epithelial-to-antigen-presenting cell (APC) transdifferentiation, amplifying autoimmune recognition; (5) Systemic immunomodulatory disruption: Chronic iodine overload may destabilize cytokine networks, activate CD4^+^ T cells, and initiate Fas/FasL-mediated apoptotic pathways in thyrocytes via sustained proinflammatory cytokine secretion (e.g., IFN-γ, TNF-α) (3).

Cytokines represent a class of low-molecular-weight signaling molecules, including both polypeptides and glycoproteins, that are produced by various cell types. These immunomodulatory mediators exert crucial biological effects by coordinating both pro-inflammatory and anti-inflammatory responses. Dysregulated cytokine production can lead to thyrocyte damage and impair normal thyroid function, thereby contributing significantly to the autoimmune mechanisms underlying GD (4). Analysis of serum cytokine profiles in treatment-naïve Graves’ disease (GD) patients provides valuable insights into cytokine network dynamics during the initial, immunologically distinct stages of disease development. Such profiling may reveal aberrant cytokine patterns that contribute to early autoimmune activation, potentially identifying novel targets for early therapeutic intervention to alter disease progression.

Previous studies have demonstrated that cytokines derived from T lymphocyte subsets can participate in the pathogenesis of GD by disrupting the balance of Th1/Th2. Specifically, Th1-related pro-inflammatory cytokines, such as IFN-γ, IL-2, and TNF-α, if overexpressed, will induce abnormal expression of HLA class II molecules on thyroid cells, which will stimulate the production of thyroid-stimulating antibodies, namely TSAb. Th2-related anti-inflammatory cytokines, such as IL-4 and IL-10, show compensatory increases. This reflects the failure of immune regulation compensation, resulting in the disruption of systemic immune homeostasis (4). One point to mention is that in the early stage of GD, there will be a characteristic cytokine surge. Compared with the healthy control group, the levels of IL-6 and IL-17 will increase by 3 to 5 times. This situation may, with the help of STAT3/RORγt-dependent transcriptional activation, jointly promote the commitment of the Th1/Th17 lineage and amplify the thyroiditis cascade response. However, the specific mechanism of action of cytokines produced by T cell subsets in iodine-induced GD hyperthyroidism and how they dynamically interact in the thyroid cytokine network have not been fully understood yet. This knowledge gap is particularly evident concerning how iodine exposure influences the temporal and spatial regulation of autoimmune effector responses.

The LEGENDplex™ multiplex immunoassay (BioLegend), a fluorescent microsphere-based analytical platform utilizing dual-antibody sandwich immunoassays coupled with flow cytometric detection, enables high-throughput quantification of multiple immune mediators across diverse species and biological specimens. Compared with conventional enzyme-linked immunosorbent assays (ELISA), this technology demonstrates superior sensitivity (detection limit <1 pg/mL), broader dynamic range (3–4 logs), and enhanced specificity through spectral deconvolution of antibody-conjugated microspheres, while conserving precious clinical samples (requiring only 25-50 μL serum per test).

Capitalizing on these technical advantages, this study employed LEGENDplex™ Human Th Cytokine Panel to simultaneously quantify 12 T cell subset-associated cytokines (including IL-2, IL-4, IL-6, IL-10, IL-17A, IFN-γ, TNF-α) in newly diagnosed GD hyperthyroidism patients stratified by WHO iodine status criteria. Systematic evaluation of cytokine correlations with SIC and thyroid function parameters (FT3/FT4/TSH/TRAb) was conducted to decipher the immunopathogenic interplay under iodine-excess conditions. These results may facilitate the development of cytokine-derived biomarkers for improved disease classification while revealing new therapeutic targets, especially for iodine-exposed patient subgroups demonstrating suboptimal responses to standard antithyroid therapies.

## 2 Study population and baseline characteristics

A total of 80 newly diagnosed GD patients and 80 euthyroid adults were prospectively recruited from the Endocrinology Clinic of the Second Affiliated Hospital of Harbin University. Comprehensive baseline data were collected, including demographic characteristics (name, sex, age), detailed GD-related medical history, and medication profiles.

### 2.1 Inclusion criteria for GD hyperthyroidism

Diagnosis was in line with the criteria stipulated in the 9th Edition of Internal Medicine, with the following requirements:

### 2.2 Essential criteria

Clinical hyperthyroidism confirmed by biochemical tests: TSH <0.56 mIU/L, FT4 >16.02 pmol/L, and FT3 >7.37 pmol/L (reference ranges according to the institutional laboratory standards). Thyroid ultrasonography reveals diffuse hyperplasia accompanied by increased vascularity, presenting the so-called “thyroid inferno” sign.

### 2.3 Supportive criteria (at least one is required)

Positive thyroid autoantibodies: TRAb >1.75 IU/L and/or TPOAb >34 U/mL. Graves’ orbitopathy, manifested as eyelid retraction, exophthalmos, or infiltrative ophthalmopathy. Dermopathy, including pretibial myxedema or digital clubbing.

### 2.4 Exclusion criteria

Participants were excluded if they met any of the following conditions: (1) Age-related exclusions (infants, children <18 years, pregnant/lactating women); (2) Severe thyrotoxic complications (thyrotoxic heart disease, thyroid storm); (3) Pre-existing organ dysfunction, decompensated cardiovascular disease, active gastrointestinal disorders); (4) Treatment-related adverse events; (5) Recent iodine exposure (iodinated contrast administration within 3 months, regular iodine-containing medications, habitual high-iodine diet, or occupational iodine contact).

### 2.5 Ethical compliance

This study protocol was approved by the Institutional Review Board of the Second Affiliated Hospital of Harbin Medical University. All participants provided written informed consent before enrollment. The investigation strictly adhered to ethical principles outlined in the Declaration of Helsinki and complied with national regulations for human biomedical research, including standardized biospecimen collection and storage protocols.

## 3 Experimental procedures

### 3.1 Serum iodine, thyroid function, and cytokine measurements

In this study, serum samples were collected from patients when the initial diagnosis of GD had not yet started treatment or when the treatment was less than one month, reducing the impact of treatment methods on serum iodine status. Peripheral venous blood (10 mL) was collected using disposable vacuum coagulation tubes. After 30 minutes of room temperature incubation, the serum was separated by centrifugation. Serum total iodine levels were determined using arsenic-cerium catalytic spectrophotometry (by Chinese health industry standard WS/T 572-2017) at the Medical Laboratory of Harbin Medical University School of Public Health. To ensure measurement consistency, all analyses were conducted by the same trained technician using identical instrumentation throughout the study. Participants were stratified into three iodine status groups per WHO criteria: excessive (>90 μg/L), adequate (45-90 μg/L), and deficient (<4 μg/L) based on SIC reference ranges (https://www.who.int/publications/i/item/9789241595827). Thyroid function parameters (TSH, FT3, FT4, TT3, TT4) and autoantibodies (TPOAb, TGAb) were analyzed at the hospital’s endocrinology laboratory center using standardized chemiluminescent immunoassays.

### 3.2 Cytokine profiling

Serum concentrations of 12 Th cell-associated cytokines (IL-2, IL-4, IL-5, IL-6, IL-9, IL-10, IL-13, IL-17A, IL-17F, IL-22, IFN-γ, TNF-α) were quantified using LEGENDplex™ Human Th Cytokine Panel (BioLegend) following manufacturer protocols. LEGENDplex™ Data Analysis Software(https://www.biolegend.com/enus/immunoassays/legendplex/support/software) was employed for spectral unmixing, standard curve generation (R²>0.99), and concentration interpolation. Values below assay detection limits (IL-6: 0.7 pg/mL; IL-17A: 0.3 pg/mL) were censored as 0 pg/mL.

### 3.3 Preparation of reagents

(1) Beads: Vortex Beads for 1min to make Beads fully mixed, dilute 13X Beads with Assay Buffer to 1X, calculate the amount of Beads, and the total number of Beads added in each well after mixing is 25ul.
(2) Wash Buffer: 20X WB was prepared into IX WB with 25ml Assay Buffer+475ml distilled water.
(3) Mitrix B: 5mL Assay Buffer was added to Mitrix B powder, thoroughly mixed and left for 15 minutes.
(4) Standard substance: 250ul Assay Buffer was added to the standard substance, fully mixed and left for 10 minutes, and diluted to different concentrations of standard substance according to the gradient (**Table 1**).
(5) Samples: The samples to be tested were thawed and equilibrated to room temperature, the serum samples were diluted twice with assay buffer (50μl sample +50μl assay buffer), and 25μl diluted samples were taken into a 1.5ml EP tube.

**Table 1:** Dilution factors for standard preparation.

Tube/Standard ID	Serial dilution	Assay buffer to add	Standard to add	Concentration
C7	–	–	–	10000
C6	1:4	75μL	25μL of C7	2500
C5	1:16	75μL	25μL of C6	625
C4	1:64	75μL	25μL of C5	156.3
C3	1:256	75μL	25μL of C4	39.1
C2	1:1024	75μL	25μL of C3	9.8
C1	1:4096	75μL	25μL of C2	2.4
C0	–	75μL	–	0

Add 250 μl of Assay Buffer to the standard sample, mix thoroughly and let it stand for 10 minutes. Then aliquot into 5 tubes of 1.5 ml EP tubes (50 μl each). Mark one of the EP tubes as the highest concentration standard sample C7. Take seven new 1.5 ml EP tubes and label them as C6/C5/C4/C3/C2/C1/C0 respectively. Add 75 μl of detection buffer to each tube. Take 25 μl from C7 and add it to C6, pipette up and down 40 times to mix well. Perform 4-fold serial dilutions successively until C1. C0 is the detection buffer with a concentration of 0 pg/ml.

### 3.4 Experimental procedures

**Figure 2:**
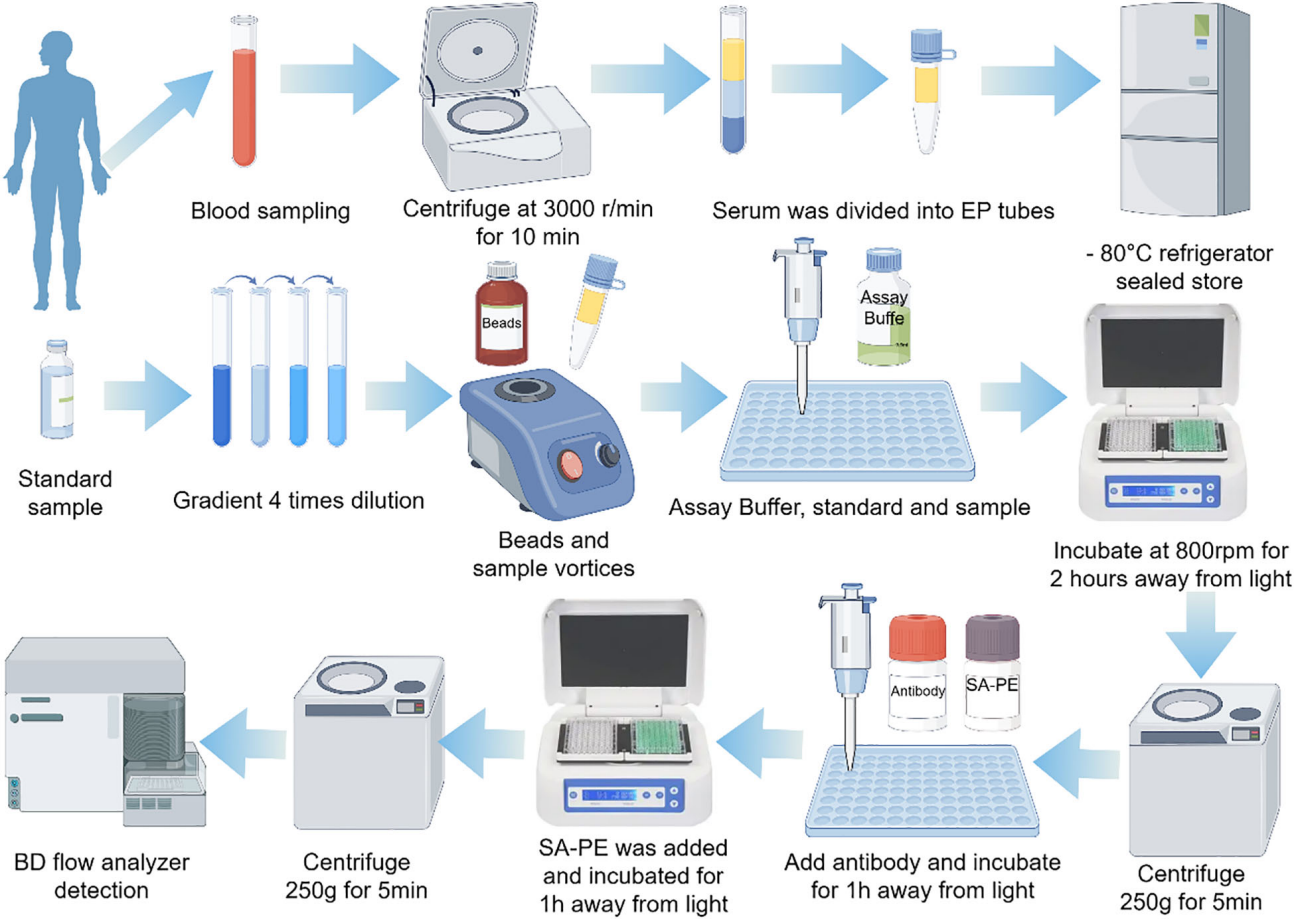
Experimental procedures. SA-PE, PE-labeled streptavidin.

(1) In 96-well plate, add the corresponding 25μl standard to the standard well, and add the corresponding 25μl sample to the sample well.
(2) Add 25μl Matrix B to standard Wells, and add 25μl Assay Buffer to each sample well.
(3) Add another 25μL of Beads to each well (vortexing Beads before adding Beads);
(4) Seal the plate with plate sealing membrane, wrap it with tin foil in the dark, and incubate at 800rpm for 2 hours at room temperature in the dark. Then, the plate is centrifuged at 250g for 5 minutes. Immediately after centrifugation, the plate is gently flipped to remove the liquid, and blue microspheres can be seen at the bottom of the hole after centrifugation.
(5) Add 200μl 1X Assay Buffer to each well, incubate for 1 minute, centrifuged at 250g for 5 minutes, add 25μl detection antibody to each well, attach the sealing membrane to the lid, shake and incubate at 800rpm for 1 hour at room temperature in the dark, add 25μl SA-PE to each well, seal with the sealing plate membrane, wrap with tin foil in the dark. The mixture was incubated at 800rpm for 30 min at room temperature with shaking, centrifuged at 250g for 5 min, and the liquid was gently shaken off.
(6) Add 200μl 1X wash solution to each well, incubate for 1 minute, seal with the sealing plate membrane, centrifuge at 250g for 5 minutes, gently shake off the liquid, add 200-400μl 1X wash solution to each well, resuspend the microspheres with a gun, transfer to a flow tube or EP tube, and prepare for the machine (**Figure 2**).

### 3.5 Template setting and data collection

**Figure 3:**
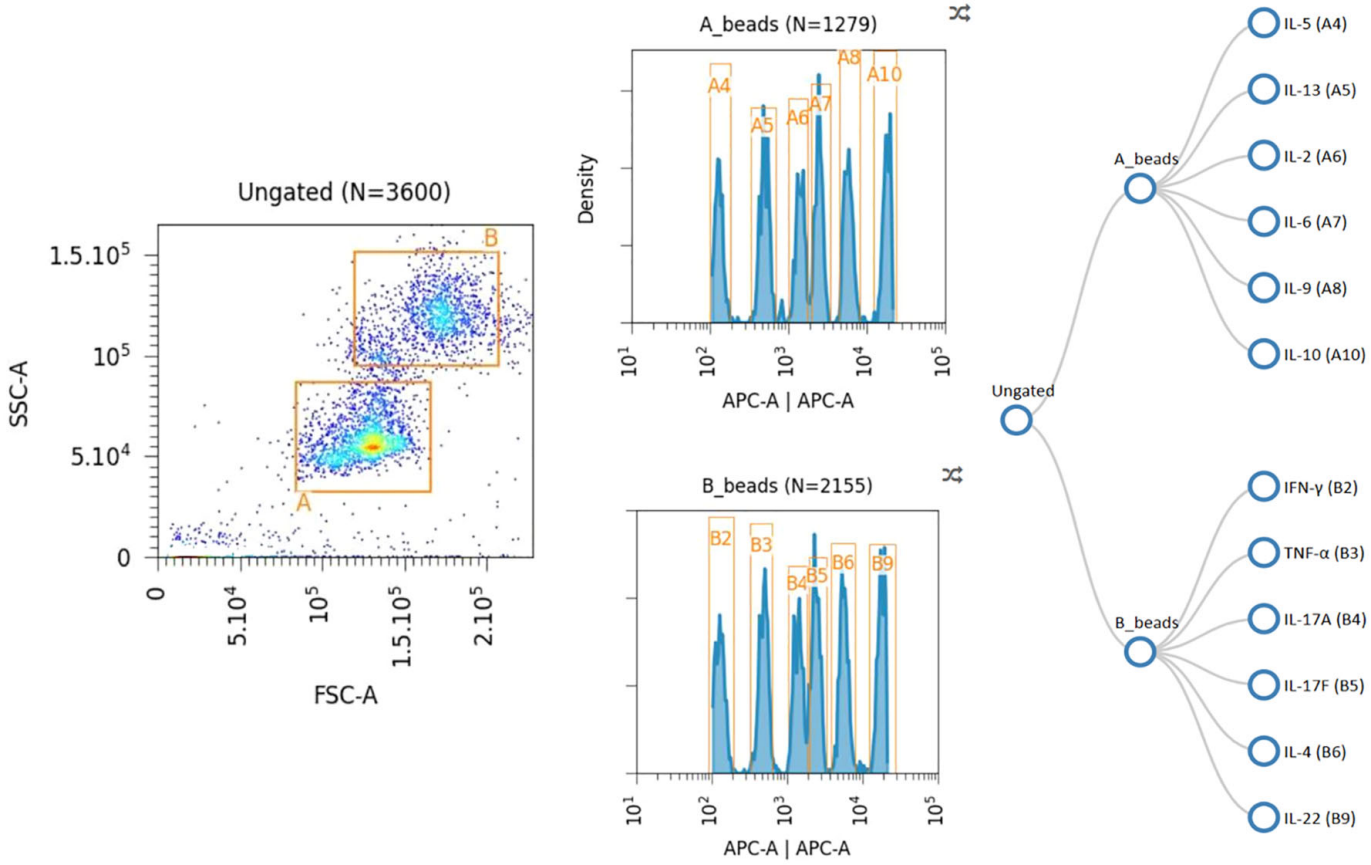
Data collection for 12 cytokines. A-beads:IL-5, IL-13,IL-2,IL-6,IL-9,IL-10;B-beads:INF-γ,TNF-α,IL-17A,IL-17F,IL-4,IL-22.

**Figure 4:**
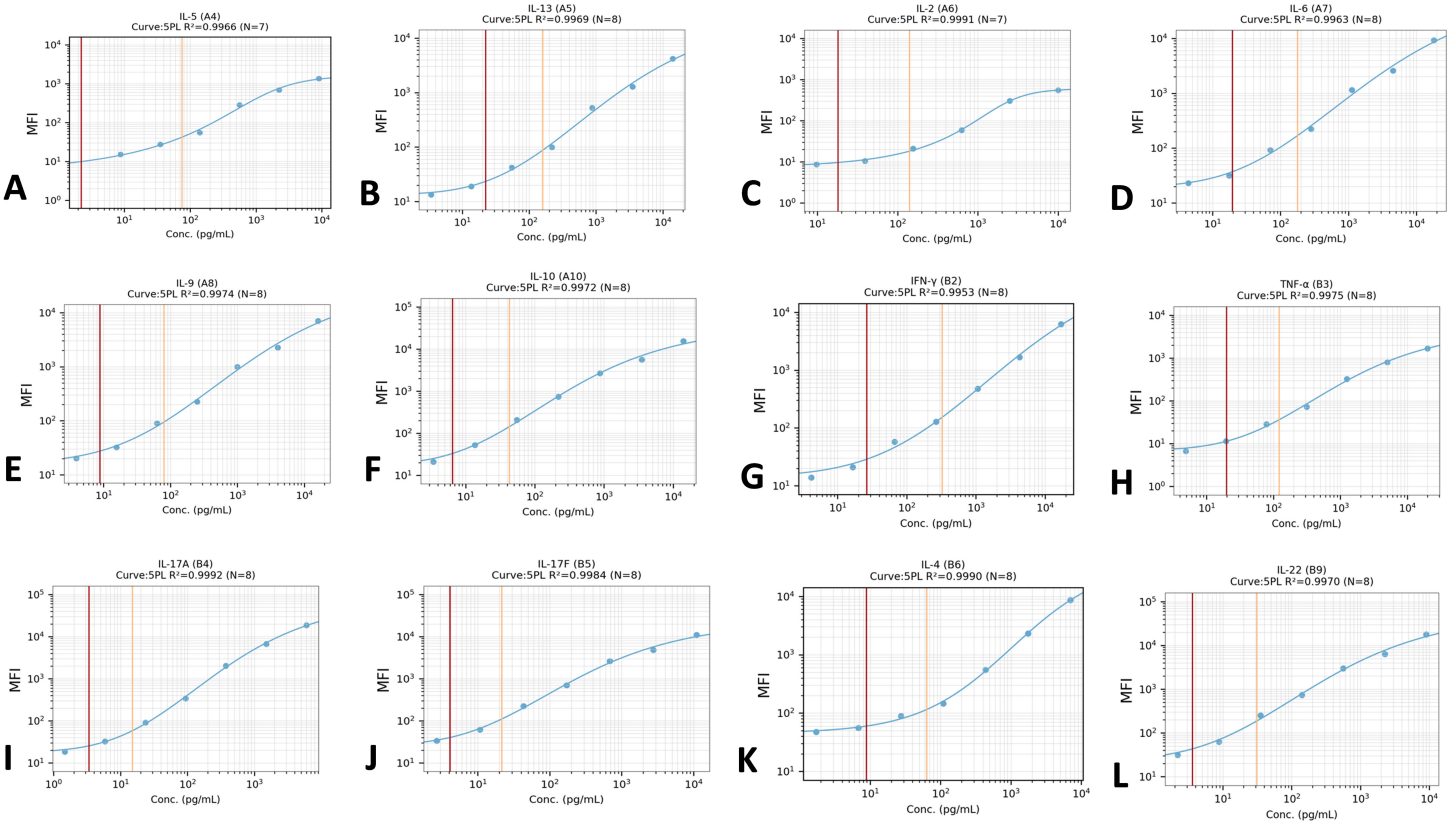
Standard curves for 12 cytokines. All standard curve generation (R²>0.99). **(A-L)** respectively represent the standard curves of IL-5, IL-13, IL-2, IL-6, IL-9, IL-10, IFN-γ, TNF-α, IL-17A, IL-17F, IL-4 and IL-22.

(1) Adjusting the PMT voltage of the flow cytometer instrument: By adjusting the voltage of the SSC and FSC channels, the two gates P1 (6 kinds of Bead A) and P2 (7 kinds of Bead B) were differentiated according to their fluorescence intensity in the APC channel by running Raw beads (**Figure 3**).
(2) The FITC channel was adjusted so that most of the APC scatter plots under gate A+B were located at 1×10^1^-1×10^2^.
(3) adjust the APC channel so that the APC fluorescence intensity of the observed population is between 10^2^ and 10^5^;
(4) The PE voltage was adjusted to make the fluorescence intensity between 10^1^-10^2^, and the PE fluorescence intensity was confirmed to be no more than 105 using C7 standard.
(5) Save the template: The principle is that the PE signal of C7 does not exceed the right edge, and C0 does not press inside the left edge. After the template is set up, the data is recorded. After the data is collected and the original file (FCS 3.0 or LMD file) is exported, Data Analysis was performed using LEGENDplex™ Data Analysis Software online (**Figure 4**).

## 4 Statistical analysis

Data Organization: Data were recorded and verified for accuracy using Microsoft Excel (2019 edition). Statistical analyses were performed using SPSS (version 22.0), and flowcharts were created using FigDraw (version 2.0). Statistical graphs were generated via the CNSKnowall research platform.

Statistical Methods: Normality Testing: Continuous variables (e.g., age, SIC, FT4, FT3) were assessed for normality using the Kolmogorov-Smirnov test. Normally Distributed Data: Variables with normal distribution were expressed as mean ± standard deviation ( $\bar{x}\pm s$ ). Comparisons between two groups were analyzed using the independent samples t-test, and comparisons among multiple groups were performed with the Kruskal-Wallis H test. Non-Normally Distributed Data: Variables such as TRAb, TgAb, and TPOAb were expressed as a median and interquartile range [M(Q1, Q3)]. Comparisons between two groups used the Mann-Whitney U test, and comparisons among multiple independent groups used the Kruskal-Wallis H test. Categorical Data: Frequency counts (e.g., number of patients with different SIC levels) were presented as percentages (%). Group differences were analyzed using the chi-square test, and comparisons of proportions between two groups were performed with Fisher’s exact test. Reference Range: The 95% medical reference range for SIC was determined using the percentile method (P2.5, P97.5). Correlation Analysis: Spearman correlation (for non-normally distributed data or nonlinear relationships) and Pearson correlation (for approximately normally distributed data to assess linear relationships) were used to evaluate associations between SIC and thyroid function indicators. P < 0.05 was considered statistically significant.

## 5 Results

### 5.1 Basic information

**Figure 5:**
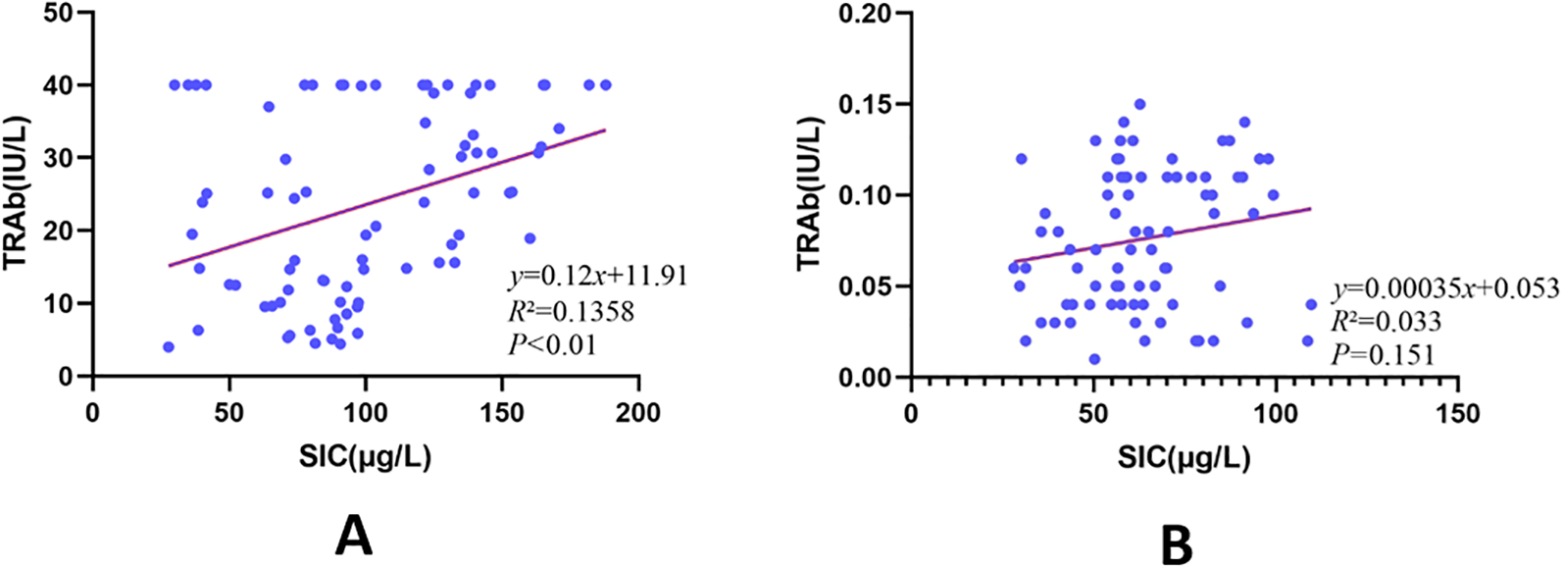
Correlation between SIC and TRAb levels in GD and NC groups. R² represents the correlation coefficient. **(A)** represents the correlation between SIC and TRAb in the GD group; **(B)** represents the correlation between SIC and TRAb in the NC group.

From the outset, inclusion in the study encompassed 80 individuals newly diagnosed with hyperthyroid GD alongside an equivalent number of healthy adults, who were participants sourced from the Endocrinology Department at the Second Affiliated Hospital affiliated with Harbin Medical University. Within this cohort—comprised of all 160 subjects—SIC assessments were conducted. Applying WHO’s international classification benchmarks (defining iodine levels as low if SIC <45 g/L, moderate within 45–90 g/L, and elevated when >90 g/L), the ultimate grouping formed was: Within the GD I faction resided 25 hyperthyroid GD patients exhibiting increased SIC (148.62 ± 17.63 g/L), inclusive of 8 males and 17 females averaging 32.63 ± 13.16 years of age; In contrast, GD II patients numbered similarly at 25, displaying moderate SIC (72.33 ± 12.08 g/L), consisting of 5 males juxtaposed with 20 females, mean aged at 37.44 ± 10.27 years; Meanwhile, comprising the NC category, 25 healthful controls with comparable SIC readings participated (75.24 ± 7.94 g/L), tallying 11 males against 14 females, with a collective mean age of 37.56 ± 9.76 years. Across the tripartite divisions, disparities in age remained statistically insignificant (P > 0.05). TRAb level evaluations unveiled marked intergroup variances (P < 0.001), wherein heightened TRAb characteristically distinguished GD I subject faces vis-a-vis those in GD II [30.57 (range: 27.01–34.14) IU/L compared to 17.05 (range: 12.08–22.01) IU/L, P < 0.001; reference **Figure 4**. Ordinary hematological examinations together with hepatic and renal function indicators adhered consistently within typical boundaries throughout all groups, devoid of discernible discrepancies between them (P > 0.05). It became evident that divergences across SIC quantities among the triadic groupings held significance (GD I: 148.62 ± 17.63 g/L, GD II: 72.33 ± 12.08 g/L, NC: 75.24 ± 7.94 g/L, P< 0.001). A correlation investigation incorporating every participant illuminated a substantial positive linkage binding SIC levels with TRAb inside GD circles (correlation coefficient r = 0.136, P < 0.001), yet notably abstained from manifesting similar correlations amid health-evident counterparts (r = 0.033, P = 0.151; see **Figure 4** for illustration) (**Figure 5**).

### 5.2 Cytokine levels

In the majority of GD patients and participants in the NC group, cytokine levels were below the lower detection limit of the multiplex assay and thus recorded as 0 pg/mL. This research indicated that serum IL-6 Concentrations were notably higher in GD patients than in the NC group. Specifically, in the GD I group, the median (interquartile range, IQR) was 94.37 (45.03–102.39) pg/mL, compared to 4.36 (1.93–4.37) pg/mL in the NC group (P < 0.001). In the GD II group, it was 83.50 (46.05–90.87) pg/mL versus 3.97 (1.86–6.08) pg/mL in the NC group (P < 0.05). Moreover, there was a significant difference in IL-6 levels between the GD I and GD II groups when stratified according to iodine nutritional status [94.37 (45.03–102.39) pg/mL vs. 83.50 (46.05–90.87) pg/mL, P < 0.05]. Likewise, serum IL-17A concentrations were substantially elevated in GD patients relative to the NC group. In the GD I group, the median (IQR) was 6.43 (3.32–7.05) pg/mL, as opposed to 0.25 (0.05–0.45) pg/mL in the NC group (P < 0.001). In the GD II group, it was 3.55 (2.28–4.83) pg/mL compared to 0.25 (0.05–0.45) pg/mL in the NC group (P < 0.05). A significant disparity in IL-17A levels was also detected between the GD I and GD II groups classified by iodine status [6.43 (3.32–7.05) pg/mL vs. 3.55 (2.28–4.83) pg/mL, P < 0.05].

Compared with the NC group, the serum concentrations of IL-17F were significantly elevated in patients with GD. Specifically, in the GD I group, the median (inter-quartile range, IQR) value was 51.69 (33.38–69.99) pg/mL, as opposed to 18.22 (9.33–27.11) pg/mL in the NC group (P < 0.05). In the GD II group, it was 50.68 (32.55–65.48) pg/mL compared to 18.22 (9.33–27.11) pg/mL in the NC group (P < 0.05). Likewise, the serum levels of IL–9 were notably higher in GD patients than in the NC group. For the GD I group, the value was 4.09 (2.57–6.02) pg/mL, while in the NC group it was 1.09 (0.38–1.80) pg/mL (P < 0.001). In the GD II group, it was 2.07 (0.99–3.16) pg/mL compared to 1.09 (0.38–1.80) pg/mL in the NC group (P < 0.05). Moreover, significant differences in serum IL-22 concentrations were also detected between GD patients and the NC group. In the GD I group, the median (IQR) was 5.69 (3.54–8.27) pg/mL, in contrast to 1.07 (0.42–1.72) pg/mL in the NC group (P < 0.05). In the GD II group, it was 3.71 (2.37–5.06) pg/mL compared to 1.07 (0.42–1.72) pg/mL in the NC group (P < 0.05). Nevertheless, when stratified by iodine nutritional status, no significant differences in IL-22 levels were found between the GD I and GD II groups (P > 0.05).

**Figure 6:**
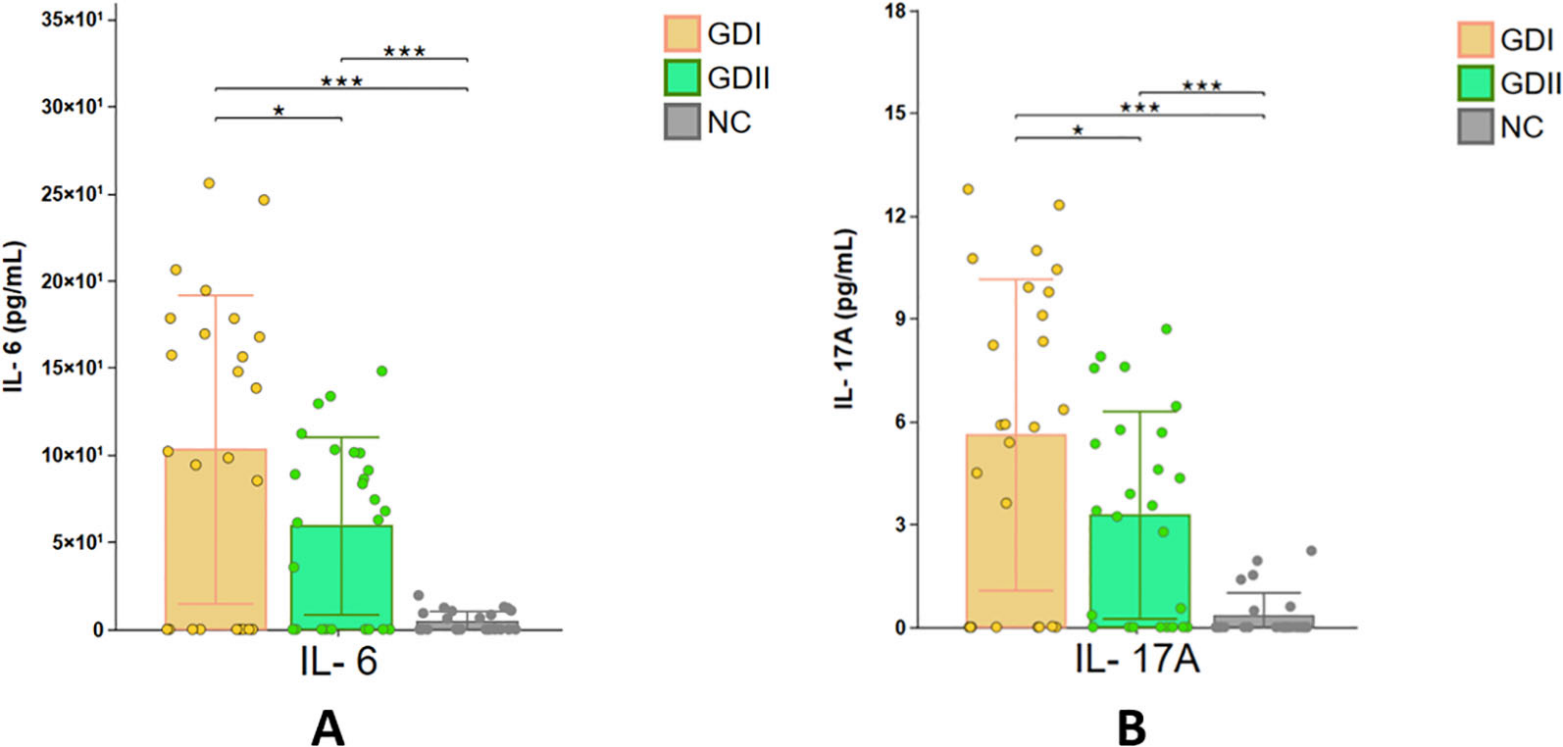
Comparison of cytokine levels among GD I, GD II, and NC groups. GD I: High iodine (HI) group; GD II: Adequate iodine (AI) group. *: p < 0.05, **: p < 0.01, ***: p < 0.001. **(A)** represents the expression levels of IL-6 in the GDI, GDII and NC groups; **(B)** represents the expression levels of IL-17A in the GDI, GDII and NC groups.

Serum concentrations of IL-4, IL-5, IL-13, IL-2, IFN-γ, and TNF-α were higher in GD patients compared to the NC group; however, these differences lacked statistical significance (P > 0.05), and no significant variations were observed across iodine nutritional status subgroups (P > 0.05). In contrast, serum IL-10 levels were lower in GD patients than in the NC group, though this difference also did not reach statistical significance (P > 0.05), with no notable disparities between iodine status subgroups (P > 0.05) (**Table 2**, **Figure 6**).

**Table 2:** Cytokine levels stratified by iodine nutritional status in GD patients and the NC group.

Detection index	GD+AI (n=25)	GD+HI (n=25)	NC (n=25)
SIC (μg/L)	148.62 ± 17.63	72.33 ± 12.08	75.24 ± 7.94
Age (years)	32.63 ± 13.16	37.44 ± 10.27	37.56 ± 9.76
FT3 (pmol/L)	25.55 ± 12.81	24.63 ± 9.04	4.69 ± 1.38
FT4 (pmol/L)	72.22 ± 25.53	48.32 (42.28,54.36)	12.79 ± 6.20
TSH (uIU/mL)	0.0065 (0.0048,0.0082)	0.014 (0.0065,0.021)	2.08 ± 0.90
TgAb (uIU/mL)	395.60 (128.13,663.07)	224.16 (87.04,361.28)	9.60 (4.29,14.92)
TPOA (uIU/mL)	492.79 (242.05,743.52)	309.84 (160.32,459.35)	7.30 (3.23,11.37)
TRAb (IU/L)	30.57 (27.01,34.14)	17.05 (12.08,22.01)	0.074 (0.059,0.90)
WBC (10^9^/L)	5.94 ± 1.22	5.29 ± 1.41	5.41 ± 1.41
NEUT (%)	3.97 ± 1.08	3.05 ± 0.94	3.67 ± 0.92
LYMPH (%)	2.33 ± 0.69	2.19 (1.88,2.49)	2.24 ± 0.81
MONO (%)	0.46 ± 0.13	0.45 (0.41,0.53)	0.44 (0.39,0.49)
HGB (g/L)	134.74 ± 9.54	133.31 ± 8.36	134.16 ± 7.62
RBC (10^12^/l)	4.72 ± 0.45	4.29 ± 0.97	5.02 ± 0.98
PLT (10^9^/L)	289.88 (276.93,302.82)	286.51 ± 39.08	297.81 ± 32.66
ALT (U/L)	24.93 ± 8.11	24.01 ± 8.81	23.53 ± 8.07
AST (U/L)	20.75 ± 4.41	20.06 ± 4.05	20.74 ± 5.50
TBIL (umol/L)	15.66 ± 3.80	14.92 ± 4.74	11.79 ± 5.13
IL-5 (pg/mL)	3.46 ± 3.20	3.14 (1.18,5.11)	1.96 (0.98,2.94)
IL-13 (pg/mL)	3.43 ± 3.25	3.41 ± 3.16	3.24 ± 2.80
IL-2 (pg/mL)	11.95 (4.58,19.32)	11.65 (4.39,18.91)	10.72 (3.43,18.02)
IL-6 (pg/mL)	94.37 (45.03,102.39)	83.50 (46.05,90.87)	4.36 (1.93,4.37)
IL-9 (pg/mL)	4.09 (2.57,6.02)	2.07 (0.99,3.16)	1.09 (0.38,1.80)
IL-10 (pg/mL)	0.25 (0.084,0.42)	0.22 (0.054,0.38)	0.39 (0.099,0.69)
INF-γ (pg/mL)	5.64 (2.09,9.18)	6.39 (2.98,9.80)	2.73 (0.99,4.47)
TNF-α (pg/mL)	2.44 (1.59,3.29)	1.64 ± 1.31	1.54 ± 1.42
IL-17A (pg/mL)	6.43 (3.32,7.05)	3.55 (2.28,4.83)	0.25 (0.05,0.45)
IL-17F (pg/mL)	51.69 (33.38,69.99)	50.68 (32.55,65.48)	18.22 (9.33,27.11)
IL-4 (pg/mL)	11.98 (5.09,18.88)	10.93 (4.64,17.23)	8.71 (4.37,13.05)
IL-22 (pg/mL)	8.37 (5.33,11.42)	6.20 (3.45,9.17)	2.43 (1.02,3.85)

The variables with normal distribution were expressed as mean ± standard deviation ( $\bar{x}\pm s$ ). Non-normal distribution variables were expressed as median and interquartile range [M (Q1, Q3)] (25th percentile, 75th percentile).

### 5.3 Correlations between cytokines, SIC, and thyroid function parameters

This study analyzed 12 cytokines in GD patients stratified by SIC. Significant differences were observed in IL-6, IL-9, IL-17A, and IL-22 levels (P < 0.05). GD patients with elevated SIC exhibited higher IL-6 and IL-17A concentrations compared to those with adequate iodine (both P < 0.05). Consequently, we focused on exploring correlations between IL-6, IL-17A, SIC, and thyroid function parameters. Cytokine levels below the multiplex assay’s detection limit in most GD patients and NC group participants were recorded as 0 pg/mL.

**Figure 7:**
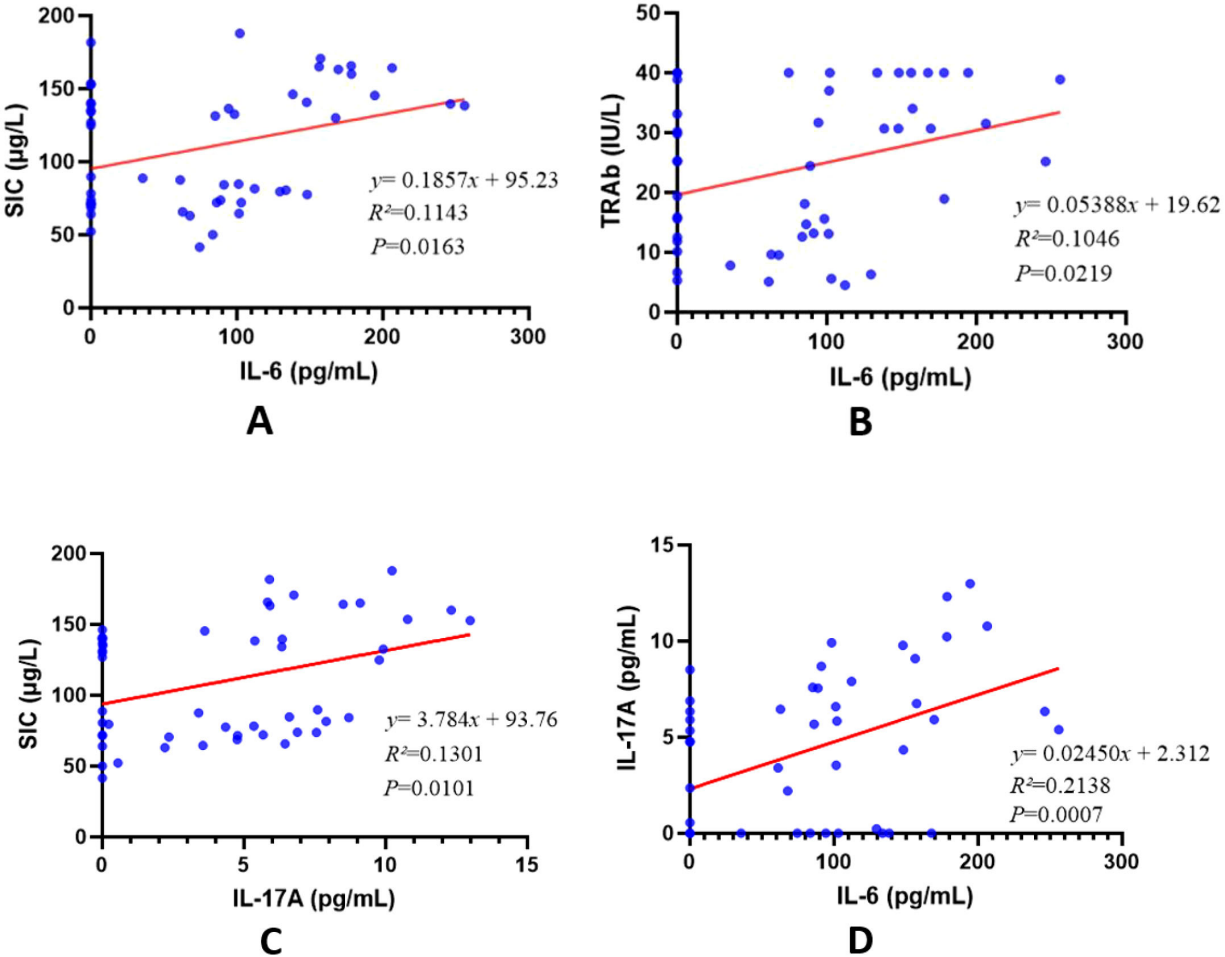
Correlation between cytokines, SIC, and TRAb. R² represents the correlation coefficient. **(A)** represents the correlation between IL-6 and SIC; **(B)** represents the correlation between IL-6 and TRAb; **(C)** represents the correlation between IL-17A and SIC; **(D)** It indicates the correlation between IL-6 and IL-17A.

IL-6 demonstrated positive correlations with SIC (r = 0.114, P = 0.016), TRAb (r = 0.104, P = 0.022), and IL-17A (r = 0.214, P < 0.001). No significant associations were observed with FT3, FT4, TSH, TPOAb, or TGAb levels (r = 0.116, 0.228, -0.024, 0.049, -0.107; P > 0.05 for all). Similarly, IL-17A showed positive correlations with SIC (r = 0.130, P = 0.010) and IL-6 (r = 0.246, P = 0.034), but no significant relationships with FT3, FT4, TSH, TPOAb, TRAb, or TGAb concentrations (r = 0.291, 0.232, -0.101, -0.078, 0.105, -0.051; P > 0.05 for all) (**Tables 3**, **4**, **Figure 7**).

**Table 3:** Correlations of IL-6 with SIC and thyroid function parameters in newly diagnosed GD patients (n=50).

Thyroid function indices	Measured values	r	P values
SIC (μg/mL)	110.31 ± 41.12	0.114	**0.016**
FT3 (pmol/L, $\bar{x}\pm s$ )	25.32 ± 10.96	0.116	0.323
FT4 (pmol/L, $\bar{x}\pm s$ )	60.43 ± 23.71	0.228	0.110
TSH (mU/L, $\bar{x}\pm s$ )	0.010 (0.006,0.014)	-0.024	0.869
TgAb [U/ml,M (Q1,Q3)]	76.42 (161.00,445.69)	0.049	0.735
TPOAb [U/ml,M (Q1,Q3)]	184.00 (274.13,560.79)	-0.107	0.458
TRAb (IU/L, $\bar{x}\pm s$ )	23.99 ± 12.47	0.104	**0.022**
IL-17A (pg/mL)	9.34 (7.00,11.69)	0.214	**<0.001**

r represents the correlation coefficient. The variables with normal distribution were expressed as mean ± standard deviation ( $\bar{x}\pm s$ ). Non-normal distribution variables were expressed as median and interquartile range [M (Q1, Q3)]. The bolded P value indicates statistical significance.

**Table 4:** Correlations of IL-17A with SIC and thyroid function parameters in newly diagnosed GD patients (n=50).

Thyroid function indices	Measured values	r	P values
SIC (μg/mL)	110.31 ± 41.12	0.130	**0.010**
FT3 (pmol/L, $\bar{x}\pm s$ )	25.32 ± 10.96	0.291	0.842
FT4 (pmol/L, $\bar{x}\pm s$ )	60.43 ± 23.71	0.232	0.876
TSH (mU/L, $\bar{x}\pm s$ )	0.010 (0.006,0.014)	-0.101	0.484
TgAb [U/ml,M (Q1,Q3)]	76.42 (161.00,445.69)	-0.078	0.589
TPOAb [U/ml,M (Q1,Q3)]	184.00 (274.13,560.79)	-0.051	0.717
TRAb (IU/L, $\bar{x}\pm s$ )	23.99 ± 12.47	0.105	0.426
IL-6 (pg/mL)	4.56 (3.22,5.44)	0.214	**<0.001**

r represents the correlation coefficient. The variables with normal distribution were expressed as mean ± standard deviation ( $\bar{x}\pm s$ ). Non-normal distribution variables were expressed as median and interquartile range [M (Q1, Q3)]. The bolded P value indicates statistical significance.

## 6 Discussion

Graves’ disease (GD) is a common organ-specific autoimmune disorder characterized by a complex pathogenesis. Current treatments fail to restore immune homeostasis fully and thus do not achieve a definitive cure. Iodine, an essential trace element required for thyroid hormone synthesis, has been unequivocally implicated in thyroid autoimmunity, with substantial evidence linking excessive iodine intake to immune dysregulation (5), Therefore, elucidating the immunoregulatory mechanisms underlying GD pathogenesis in the context of high iodine exposure and developing etiology-targeted therapies may offer novel avenues for achieving a definitive cure for GD.

Recent studies have demonstrated that cytokines play critical roles in the pathogenesis, progression, and clinical outcomes of GD. Preclinical evidence suggests that excessive iodine intake induces thyroid follicular cell injury, apoptosis, and necrosis in animal models (6). Building on these preclinical insights, this clinical study investigated dynamic changes in cytokine levels among newly diagnosed hyperthyroid GD patients stratified by iodine nutritional status. Furthermore, we performed correlation analyses with TRAb—a core antibody pivotal to disease progression, severity, and extrathyroidal manifestations in GD to elucidate the pathogenic mechanisms underlying high-SIC GD and identify novel immunotherapeutic targets.

Existing research has elucidated key immunological pathways through which excessive iodine exposure exacerbates GD. These studies have consistently shown that supra-physiological iodine intake leads to a significant elevation of TRAb while potentiating both T-lymphocyte and B-lymphocyte responses in affected patients (6). These data indicate that excessive iodine exposure may initiate TRAb production in genetically predisposed individuals via immune system dysregulation, consequently accelerating GD pathogenesis. In this study, SIC differed significantly among the three groups: GD I (high iodine: 148.62 ± 17.63 μg/L), GD II (moderate iodine: 72.33 ± 12.08 μg/L), and NC (75.24 ± 7.94 μg/L) (*P* < 0.001). Correlation analysis of all 160 participants revealed a strong positive association between SIC and TRAb levels in newly diagnosed GD patients (r = 0.369, *P* < 0.001). Notably, TRAb levels were markedly higher in the GD I group compared to GD II [30.57 (27.01–34.14) IU/L vs. 17.05 (12.08–22.01) IU/L, *P* < 0.001]. These results indicate that iodine excess significantly amplifies serum autoantibody levels in GD patients. Mechanistically, this phenomenon may involve iodine-induced activation of T-cell and B-cell responses, enhanced monocyte-to-macrophage differentiation, and disruption of immune homeostasis.

IL-6, a Th2-type cytokine with paracrine and autocrine properties, is primarily produced by monocytes and T lymphocytes. It facilitates B-cell proliferation and differentiation, mediates humoral immune responses, and broadly regulates thyroid cell growth, differentiation, and homeostasis (7, 8). Previous studies have shown elevated serum IL-6 and soluble IL-6 receptor concentrations in 31 newly diagnosed GD patients during the active phase of the disease (9). In a cohort of 118 GD patients stratified by disease severity, IL-6 levels increased proportionally with worsening clinical status, suggesting its potential as a biomarker for GD progression (10). Elevated IL-6 levels in newly diagnosed or relapsed GD patients, which decline during treatment, further underscore its role in disease pathogenesis and resolution (11). Additionally, GD patients in regions with varying iodine exposure exhibited significantly higher IL-6 levels compared to controls (*P* < 0.05) (11). In this analysis of newly diagnosed GD patients, serum IL-6 concentrations were markedly higher than in the NC group. Additionally, GD patients with high SIC had significantly higher levels of IL-6 as compared to those with enough iodine (P < 0.05). Thyroid cells from GD patients secrete IL-6 in population-based studies, and a sodium iodide stimulus further increases IL-6 secretion and damage to thyrocytes. These findings are corroborated by animal models; NaI-treated mice exhibit elevated serum thyroglobulin antibodies and IL-6 levels (7, 12). Evidence suggests that altered iodine transport by GD thyroid cells may predispose them to iodine-induced damage and cytokine release. Taken together, our data suggest that excess iodine exacerbates hyperthyroidism by increasing IL-6 levels. The concentration of IL-6 becomes normal with control of symptoms in patients with GD. Previous studies found negative correlations between the levels of IL-6 and TSH and positive correlations with FT3 and FT4 (10). This study aimed to investigate the association between IL-6 and thyroid function parameters. TRAb can be detected in almost all patients with GD and is an autoantibody specific to GD. The relationship with dysthyroidism was confirmed by a correlation of TRAb with IL-6 (r= 0.105, P = 0.022). Thus, IL-6 may induce dysthyroidism in GD. On the one hand, levels of IL-6 were positively associated with TRAb and IL-17A levels. The impact of elevated iodine levels on IL-6, TRAb, and IL-17A in GD may bring significant biological effects through multiple mechanisms. The pro-inflammatory role of IL-6: IL-6 is produced by thyroid cells and by infiltrating immune cells (e.g., macrophages, Th2 cells) and is capable of activating autoreactive T cells and B cells. In an environment high in iodine, thyroid cells may release more IL-6, which can augment this toward the next stage (13). Studies have shown a strong correlation between serum IL-6 levels and TRAb in GD patients. This suggests that IL-6 may worsen hyperthyroid symptoms through B-cell differentiation and antibody production, ultimately augmenting the pathogenic influence of TRAb. The dual effect of high iodine. Iodine is a substrate for thyroid hormone formation. But at high doses, Iodine may block the secretion of said hormone. For example, T4 accumulation increased after inorganic iodine in mice from China. However, high iodine can also stimulate IL-6 release, creating a local inflammatory milieu. The concurrent presence of hyperthyroidism and tissue destruction within the thyroid may be due to this dual mechanism in people suffering from GD (13). Association with metabolic dysregulation: In patients with GD, IL-6 has been positively associated with TRAb, and high iodine may disrupt thyroid hormone feedback regulation through IL-6, which may worsen the hypermetabolic symptoms (e.g., heat intolerance and weight loss) (14). The imbalance of Th17/Treg in the presence of IL-6 promotes Th17 cells at the expense of Treg’s function. Excess iodine can worsen this imbalance through IL-6 upregulation, compromising immune tolerance and promoting autoimmune progression of GD (13). Clinical implications of IL-6-high iodine correlation in GD: The positive correlation between IL-6 and high iodine may be an indicator of GD activity and prognosis. When assessing the possible effects of iodine in disease progression, inhibitory effects of high iodine on the IL-6 signaling pathway (eg IL-6 receptor blocker) or curbing high iodine intake may be novel adjuvant therapeutic strategies for GD (15).T This IL-6-high iodine interaction is an example of how environmental factors (iodine) can interact with immune-inflammatory reaction in the pathogenesis of GD. Disease progression worsens through inflammation promotion, autoimmune destruction exacerbation, and dysregulated metabolic worsening by this synergistic relationship. These findings broaden our understanding of the intricate mechanisms underlying GD and may guide personalized treatment strategies.

IL-17A is an important proinflammatory cytokine produced by Th17 cells, attracting neutrophils and monocytes, activating inflammation, and inducing the secretion of some important inflammatory factors such as IL-6, TNFα, etc. Abnormal IL-17 levels were supposed to play an important role in autoimmune diseases through different processes such as adhesion, recruitment, cell proliferation, and inflammation. The latest data showed that the level of IL-17 concentrations was not only increased in AITD but also specifically in GD; high value was shown between sera of the patient versus control at P < 0.01 threshold significance, and especially high values were shown among serological samples drawn before diagnosis for patients when compared to inactive cases, the latter group represented patients who are still suffering the disease on an active stage despite therapy application, which confirmed the previous statement because they are actively working against abnormal antigen presentation by thyrocyte without cure or any specific symptom’s appearance (P< 0.05) (16). Other observations found that: Serum IL-17 increases significantly, the relative amount of both mRNA and protein is markedly higher in GD than healthy subjects using human ELISA tests detecting anti-cytokines’ responses (17). Animal experiments confirm mentioned results finding elevation in the quantity of T-CD4+, CD4+, and increase IL 17 positive percentage of peripheral mononuclear population with augmented proportion of this type (Th 17) over regulatory populations (Tregs); along-with augmentation in IL -17/A at both transcripts level with parallel changes observed with cellular fraction analysis of immunocompetent animal system which was done after initiation of treatment by triiodothyronine (18). IL-17 serum level in high and middle IGD groups was obviously up-regulated, more than NC group (detectable by a few persons), also exhibited a significant statistical difference between the three groups (P<0.01). IL-17 would exert function to the pathogenesis of AITD by many ways: caused thyroid follicular cell produced more proinflammatory cytokine such as IL-6/IL-8/ICAM-1 etc., so that keeps synthesizing triiodothyronine constantly and inflammation fibrosis occurring; raised thyrocytes surface MHCA antigens contents as stimulating neuters’ population and matured for maintaining immune disorder through coordinating working with T cells activated, respectively; displayed cooperative inflamation stimulating effect on local site with IL-1b and TNF-a to amplify injury extent and tissues damaged. As to relation to thyroid function indexes, according to studies in China, it is observed that there exists a positive correlation between serum/plasma IL-17 content and TSAb in 27 cases of newly diagnosed primary GD patients (r=0.7101, P<0.0001) (19). Others have shown IL-17 is inversely related to TSH and directly related to FT3, FT4, and TRAb. In agreement, we observed serum IL-17A levels positively associated with TRAb but not correlating with TPOAb, TGAb, TSH, FT3, and FT4 in hyperthyroid GD subjects at high iodine areas; therefore, it seems more the thyroid hormone (FT3/FT4) stimulation higher the IL-17. Due to the availability of anti-Thyroid Autoimmunity as a basic immunologic decision governing most clinical decisions, we think this spectacular direct association between IL-17-TRAb provides fascinating information suggesting that IL-17 is a beneficial drug therapy. Data coming from animal models further supports evidence; nonobese diabetic H2(h4) mice exposed to Iodine showing increased splenic and thyroid Th17 cells, while IL-17 KO significantly attenuates thyroiditis development in experimental murine models, supporting the use of Th17 treatment for disease (7, 20). Clinically, refractory GD patients show higher peripheral Th17 cell proportions than those in remission (*P* < 0.05) (21). Excess iodine may promote thyroid hormone-specific antibody production and Th17 infiltration (3). Suggesting Th17 modulation as a novel therapeutic strategy for GD patients with elevated SIC. Notably, IL-6 and IL-17A levels are elevated in GO orbital tissues (22). IL-6 drives Th17 differentiation via IL-21/IL-23 pathways, activating acute-phase responses and complement cascades to amplify immune reactions (23, 24). Moreover, we also found out there was a Significant Positive Correlation Between the Concentration of Serum IL-17A and IL-6 Amongst GD Patients (r=0.214, P < 0.001), Where They Are Higher in High SIC Cases Than Others (P<0.05). There was a positive relation between a high iodine situation and high concentrations of the IL-17A involved Immune response abnormalities like abnormal inflammation during the pathological development of GD. The high Iodine promoted immune cells’ activation through different manners by expressing itself to self-Antigen expression from Thyroid Follicle Cell by Multifaceted ways: firstly Due to the Increased Immunocyte cell’s activity due to the presence of high concentration of iodine, Then due to the presentation of Antigen- Presenting Cell Dendritic cells as well as Macrophages for activated B-cells which results into induced TH4+ T-cell activation which caused the mature Pro-inflammatory population of Lymphocytes with CD4+ Th17 or more commonly known as called Th2 Cells, where up regulation of RORyt resulted in secretion of IL-17A which Promoted the differentiation of these Cytokines secreting T-helper immunecellular populations toward its proper compartmentalization state (25). IL-17A stimulates thyroid epithelial cells and fibroblasts to produce inflammatory mediators such as IL-6, establishing a positive feedback loop that exacerbates local inflammation and thyroid tissue damage (26, 27). Hypersecretion of thyroid hormones: IL-17A enhances the sensitivity of thyroid cells to TRAb by activating NF-κB and MAPK pathways, and promotes the excessive synthesis and release of thyroid hormones (T3/T4). Direct IL-17A stimulation of Graves’ disease thyrocytes resulted in abnormal proliferation and hyperfunction. Elevated iodine levels may potentiate thyroid hormone secretion through dual mechanisms: (1) upregulation of TSHR expression, and (2) synergistic amplification of cAMP-mediated signaling with IL-17A.In addition, iodine excess may also activate the inflammasome through the oxidative stress pathway and indirectly promote the production of IL-17A (28, 29). Therapeutic challenges: IL-17A-induced inflammatory signaling has “autoactivated” mechanisms (such as the SHP2-ACT1 pathway), and even the use of anti-IL-17 antibodies may have limited efficacy due to the persistence of the signal, suggesting that targeting IL-17A downstream molecules (such as SHP2) or combined with iodine intake control may be a more effective strategy in a high iodine environment (30). Iodine intake management: For GD patients in high iodine areas, limiting iodine intake may help to reduce IL-17A-mediated inflammatory response and improve the efficacy of antithyroid drugs such as methimazole. The Potential of integrated traditional Chinese and Western medicine: Traditional Chinese medicine can inhibit the IL-17A signaling pathway, reduce thyroid cell proliferation and hormone secretion, and provide a complementary treatment option for refractory GD (31).The positive correlation between high iodine and IL-17A in GD not only reflects the interaction between environmental factors and immune disorders but also reveals the core mechanism of disease progression: jointly promoting thyroid autoimmune injury through Th17 cell polarization, inflammatory signal amplification, and hormone secretion disorder. Future investigations should aim to: (1) establish precise threshold values for iodine intake in disease modulation, and (2) identify novel therapeutic targets within the IL-17A signaling cascade for potential clinical intervention.

The changes in serum IL-2 levels in patients with GD remain controversial: some studies have demonstrated a significant elevation in serum IL-2 levels among GD patients, suggesting a close association with the onset and progression of GD (32). Other studies have reported no significant difference in serum IL-2 levels between GD patients and healthy controls (31); which is consistent with our experimental findings. The role of IL-4 in the pathogenesis of Graves’ disease (GD) remains controversial. Some studies suggest that local IL-4 expression in the thyroid gland may mitigate GD progression, whereas others have observed significantly reduced plasma IL-4 levels in GD patients. In the present study, no significant difference was found in serum IL-4 levels between GD patients and healthy controls (32). Serum IL-5 levels were higher in GD patients than in healthy controls, but the difference was not statistically significant (33). This suggests that elevated IL-5 levels may be a consequence rather than a cause of GD pathological changes. Serum IL-9 levels were significantly elevated in newly diagnosed GD patients, with higher IL-9 mRNA expression in PBMCs compared to healthy controls. Increased expression of both IL-9 mRNA and protein was observed in peripheral blood. Some studies have also reported elevated IL-9 in iodine-induced autoimmune thyroiditis models (34, 35). However, the role of IL-9 in GD patients with high iodine levels remains unclear. Serum IL-10 levels, along with IL-10 mRNA and protein expression, were significantly elevated in GD patients (36). However, our study found no significant differences in these parameters among GD patients with high iodine exposure. Although IL-13 has been shown to inhibit the production of proinflammatory cytokines and regulate immune function, and is expressed in the majority of GD tissue samples (37). Analysis of serum samples showed no notable changes in IL-13 concentrations in treatment-naïve GD patients presenting with hyperthyroidism. The mechanistic role of IL-22 appears complex. Current evidence demonstrates significantly increased IL-22 mRNA and protein expression in PBMCs, along with elevated serum IL-22 levels in treatment-naïve GD patients compared to controls (20, 38). Nevertheless, limited data exist regarding IL-22’s involvement in hyperthyroidism development under high-iodine conditions. The relationship between TNF-α and autoimmune thyroid disorders remains contentious. Some studies report comparable TNF-α levels between GD patients and controls, whereas others document elevated concentrations in GD cohorts (38–40). Although post-therapeutic reductions in serum TNF-α have been documented in GD patients with hyperthyroidism, our data showed no such differences (4). Similarly, IFN-γ findings exhibit inconsistency—reports indicate decreased levels in GD patients with ophthalmopathy, while high iodine exposure may potentially influence hyperthyroidism progression through elevated serum IFN-γ and TNF-α levels (11, 38). Our study observed numerically higher (though statistically insignificant) IFN-γ and TNF-α levels in treatment-naïve GD patients with high iodine exposure compared to normal controls (NC). These divergent results may reflect methodological variations in sample collection or processing.

Our study has several limitations that should be acknowledged. First, the limited number of samples analyzed in this study led to low cytokine detection frequencies and modest correlation coefficients, indicating that expanded research with more participants is needed in subsequent investigations. Second, we did not assess the quantity of circulating Th17 cells in PBMC cultures, which could provide valuable insights into the interactions between immune components. Finally, while current therapeutic approaches for GD are well-established, they remain suboptimal. The therapeutic potential of boosting immunotherapy, especially by specifically blocking IL-6 and IL-17A production as an innovative approach for GD, needs further confirmation in future research.

## 7 Conclusions

1. Serum cytokine levels (including IL-6, IL-9, IL-17A, IL-17F, and IL-22) exhibited significant differences between healthy subjects and patients with newly diagnosed hyperthyroid GD under varying SIC. 2. In newly diagnosed hyperthyroid GD patients, serum IL-6 demonstrated Weak correlations with SIC, TRAb, and IL-17A (all *P* < 0.05), while IL-17A showed Weak correlations with SIC and IL-6 (*P* < 0.05). 3. In GD patients with elevated SIC, cytokines IL-17A and IL-6 may contribute to pathogenic processes in hyperthyroid GD.

## Data Availability

The original contributions presented in the study are included in the article/supplementary material. Further inquiries can be directed to the corresponding author.
